# Predicting early functional outcomes in aneurysmal subarachnoid hemorrhage in endovascular coiling and surgical clipping

**DOI:** 10.3389/fneur.2025.1466188

**Published:** 2025-05-07

**Authors:** Liang Chu, Kan Cao, Kuan Jiang, Yunpeng Lu, Ming Qi, Da Wu

**Affiliations:** ^1^The Affiliated Yixing Hospital of Jiangsu University, Yixing, China; ^2^The Affiliated Zhenjiang First Hospital of Jiangsu University, Zhenjiang, China

**Keywords:** aneurysmal subarachnoid hemorrhage, early functional outcomes, endovascular coiling, surgical clipping, D-dimer, nomogram

## Abstract

**Objectives:**

This study aimed to evaluate early functional outcomes in patients with aneurysmal subarachnoid hemorrhage (aSAH) treated with either endovascular coiling or surgical clipping and to develop predictive models tailored to each treatment modality.

**Materials and methods:**

Patients diagnosed with aSAH were retrospectively enrolled from two hospitals in China between January 1, 2015, and December 31, 2022. Based on the treatment approach, patients were divided into two groups: endovascular coiling and surgical clipping. Independent risk factors were identified using least absolute shrinkage and selection operator (LASSO) regression followed by multivariate logistic regression. The relative contribution of each significant factor was calculated, and nomograms were constructed accordingly. Model performance was subsequently assessed through validation analyses.

**Results:**

Multivariate analysis identified Hunt–Hess grade, Glasgow Coma Scale (GCS) score, modified Fisher Scale (mFS), D-dimer, age, and body temperature as independent predictors of early functional outcomes following endovascular coiling (all *p*-values <0.05). For surgical clipping, Hunt–Hess grade, GCS score, mFS, and D-dimer emerged as significant predictors (all *p*-values <0.05). The calculated relative contributions for endovascular coiling were 32.78% (Hunt–Hess grade), 31.99% (mFS), 4.63% (GCS score), and 13.73% (D-dimer); for surgical clipping, these values were 33.55, 38.02, 8.44, and 19.99%, respectively. Nomograms were developed for both treatment groups, and their performance was validated using receiver operating characteristic (ROC) curves, calibration plots, and decision curve analysis (DCA), demonstrating strong discriminative ability and clinical applicability.

**Conclusion:**

This study developed predictive nomogram models for early functional outcomes of aSAH patients undergoing endovascular coiling or surgical clipping treatments, emphasizing the importance of scoring systems and clinical parameters (such as D-dimer), demonstrating strong clinical utility.

## Introduction

Aneurysmal subarachnoid hemorrhage (aSAH) is a life-threatening cerebrovascular disorder with significant global morbidity and mortality. The global incidence of aSAH is approximately 6.1 cases per 100,000 person-years (1), while in China, it is estimated at around 2.0 cases per 100,000 person-years (2). Notably, epidemiological studies have established a positive correlation between aSAH incidence and advancing age (3). As populations worldwide age, the number of individuals at risk of aSAH is expected to rise, underscoring the urgent need for effective diagnostic and treatment strategies.

For patients with ruptured aneurysms in anterior or posterior circulations, endovascular coiling generally yields better short-term outcomes compared to surgical clipping (4, 5). However, surgical clipping remains the preferred approach for patients with large intracerebral hematomas or middle cerebral artery aneurysms due to its unique advantages (6). Findings from the International Subarachnoid Aneurysm Trial (ISAT) (7) showed that the endovascular coiling group had a 7% lower 1-year mortality rate than the surgical clipping group, albeit with an increased long-term risk of rebleeding.

Current clinical evaluations of postoperative outcomes after surgical clipping and endovascular coiling predominantly rely on the Hunt–Hess grading scale and the Glasgow Coma Scale (GCS). While valuable, these traditional assessment tools do not comprehensively account for individualized clinical parameters that may significantly influence patient prognosis (8). This study aimed to develop novel nomogram models based on commonly used clinical scoring systems and selected clinical parameters to assess early functional outcomes—such as neurological recovery and activities of daily living—in patients undergoing the two different surgical approaches.

## Materials and methods

### Study population

This study retrospectively and continuously collected patients with aSAH who were hospitalized and treated at the Affiliated Yixing Hospital of Jiangsu University and the Affiliated Zhenjiang First Hospital of Jiangsu University from 1st January, 2015 to 31st December, 2022. Diagnosis, surgery, and perioperative management strictly adhered to the latest clinical practice guidelines of the Chinese Medical Association (9). The surgical approach for each case was determined by a neurosurgical team of senior neurosurgeons, based on individual patient characteristics, aneurysm features, and in accordance with available clinical guidelines. When both surgical options were deemed feasible, the final decision was made through team consensus, taking into account the preferences of the patient’s family or designated surrogate decision-maker. Postoperatively, patients typically required intensive care management, which followed guideline-based protocols and included close monitoring, appropriate sedation and analgesia, cardiovascular support, vasospasm prevention, and active management of potential complications. The study adhered to the Helsinki Declaration and obtained approval from the ethics committees of two hospitals, with approval numbers 2023043 and K-20230086-w. All participants or their authorized representatives were required to sign an informed consent form during hospitalization, ensuring their full understanding of the purpose, methods, risks, and benefits of the surgery and study, and indicating their voluntary participation.

Inclusion criteria include: (1) age 18 years or older; (2) confirmed as aSAH through laboratory and imaging examinations; (3) patients must undergo either endovascular coiling or surgical clipping treatment. Exclusion criteria include: (1) previous hospitalization for aSAH; (2) subarachnoid hemorrhage caused by other reasons, including vascular malformation, cerebral atherosclerosis, trauma, etc.; (3) the presence of severe cardiovascular, liver, kidney or other vital organ diseases; (4) loss of clinical parameters such as platelet count and D-dimer.

### The study variables and outcomes

The study variables include demographic information (age and sex), physical examination data (body temperature and mean arterial pressure), laboratory tests (D-dimer, and platelet count), imaging examinations (length, width, height, position, etc.), past medical history (hypertension and diabetes), clinical scoring tools (GCS, Hunt–Hess, and mFS), treatment modalities (endovascular coiling and surgical clipping), and the use of stents. Patients were stratified into the endovascular coiling group and the surgical clipping group based on treatment methods.

The primary endpoint of this study was the early functional outcomes of patients 1 month after surgery. Considering that the modified Rankin Scale (mRS) is recommended for assessing neurological recovery status, this study utilized the mRS score at 1 month post-surgery as the primary endpoint, with scores ranging from 0 to 3 indicating a favorable prognosis and scores from 4 to 6 indicating an unfavorable prognosis (10).

### Statistical analysis

Chi-square tests were used for categorical variables, while non-parametric tests were employed for continuous variables. Univariate and multivariate logistic regression analyses were conducted to determine independent predictive factors and calculate their relative weights on the outcome variable. Two separate nomogram models were developed to predict early functional outcomes in aSAH patients undergoing endovascular coiling and Surgical clipping, respectively. Receiver operating characteristic curves (ROC) were plotted, and the area under the curve (AUC) was calculated to assess the discriminative ability of the nomogram. Calibration curves were generated to illustrate the consistency between predicted probabilities and observed outcomes across various predicted probabilities. Decision curve analysis (DCA) was conducted to evaluate the clinical utility of the nomogram. Finally, risk stratification was performed. All statistical analyses were conducted using SPSS version 26.0 and R version 4.3.1, with statistical significance defined at the conventional threshold of *p* < 0.05 (two-tailed).

## Results

### Characteristics of patients in the endovascular coiling and surgical clipping groups

**Figure 1:**
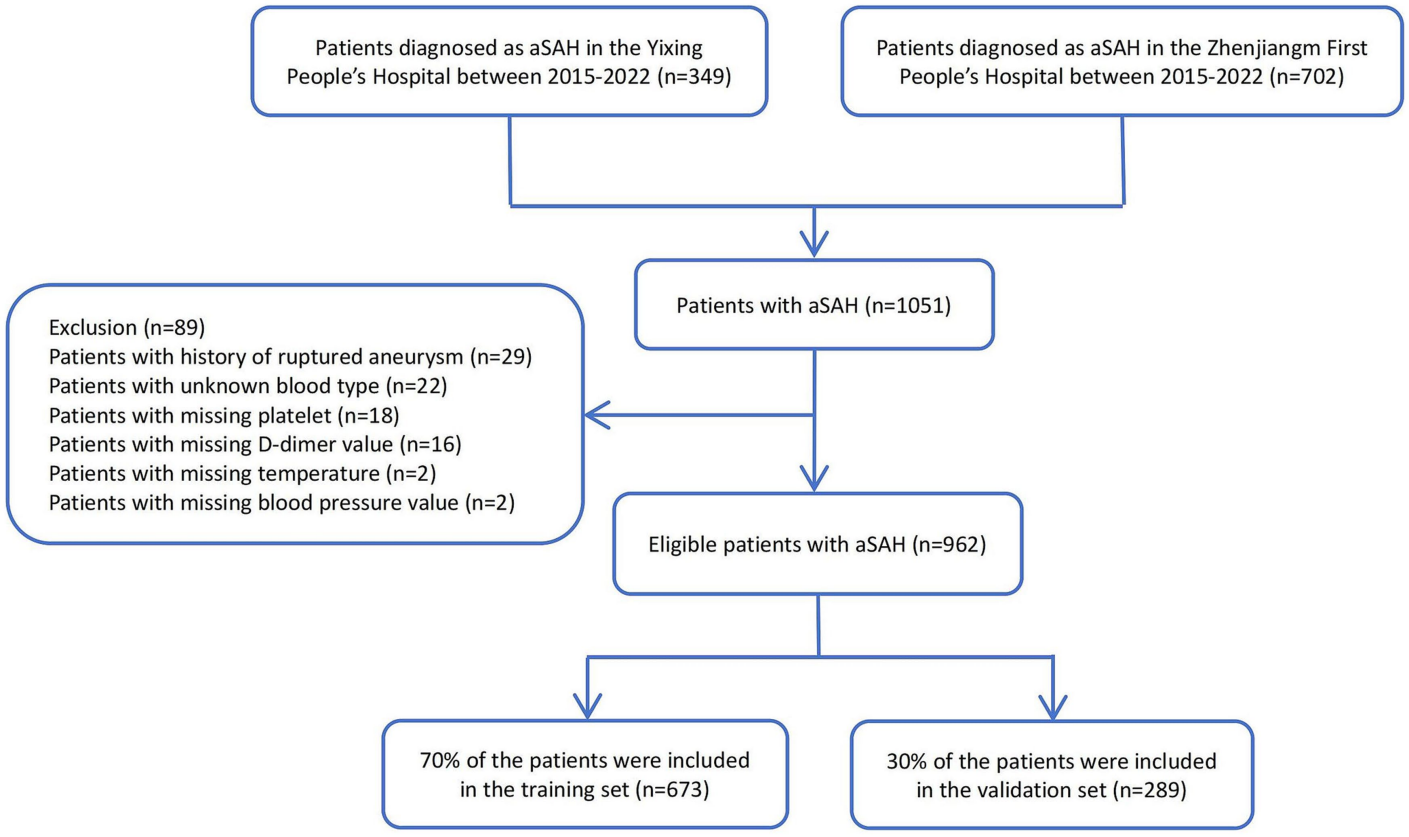
Flow diagram of eligible patients diagnosed with aSAH.

**Figure 2:**
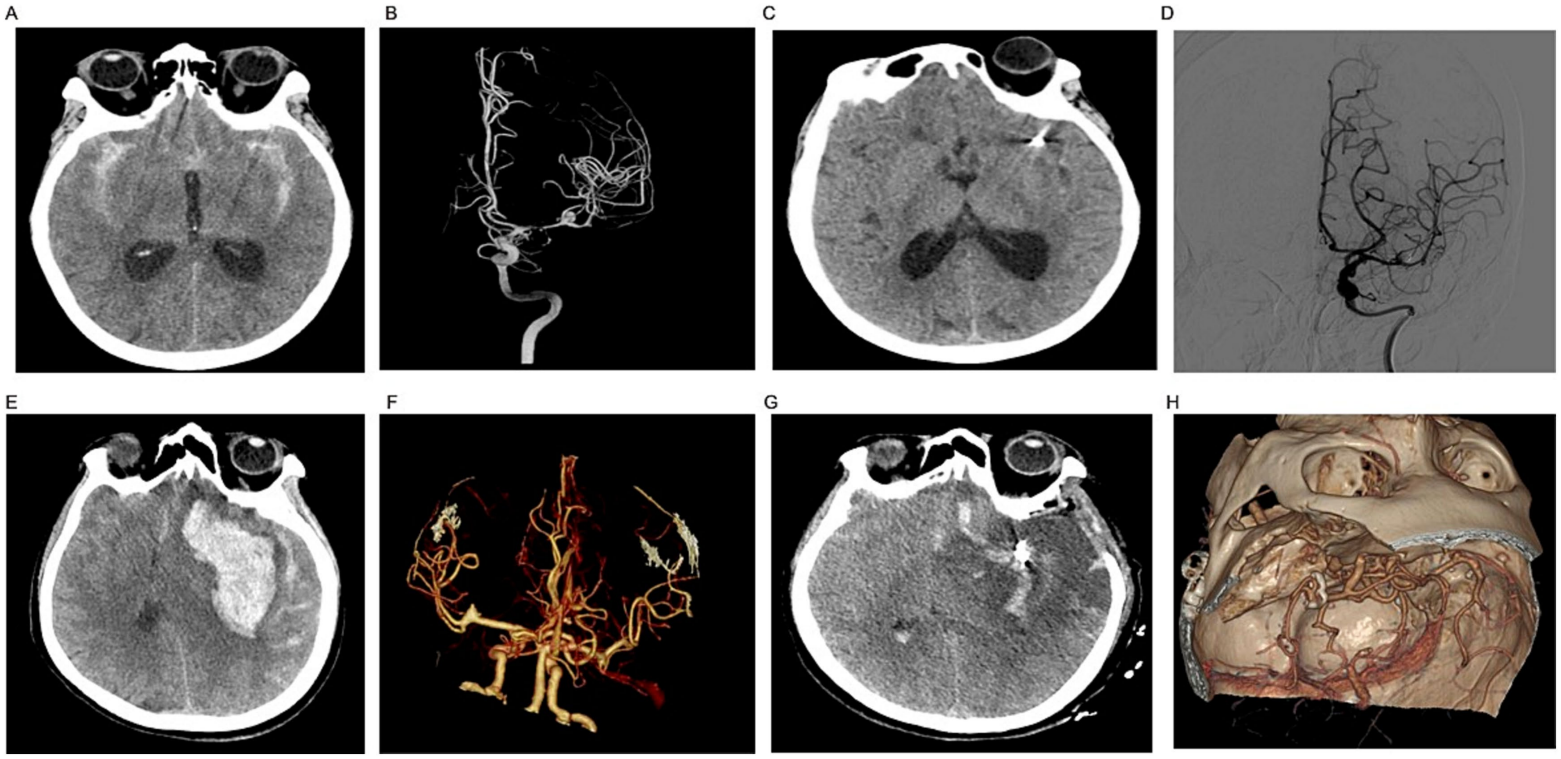
**(A)** Ruptured aneurysm of the left middle cerebral artery with subarachnoid hemorrhage. **(B)** DSA showing left middle cerebral artery aneurysm. **(C)** Post-embolization CT of left middle cerebral artery aneurysm. **(D)** Post-embolization angiogram of left middle cerebral artery aneurysm. **(E)** Left cerebral middle artery aneurysm rupture with subarachnoid hemorrhage and temporal lobe hematoma. **(F)** Left cerebral middle artery aneurysm CTA imaging. **(G)** Post-clipping of left cerebral middle artery aneurysm and clearance of temporal lobe hematoma. **(H)** Post-clipping of left cerebral middle artery aneurysm.

A total of 962 patients diagnosed with aSAH were included in the final analysis cohort (Figure 1). Overall, the proportion of early adverse outcomes in the surgical clipping group (32.7%) was higher than that in the endovascular coiling group (15.0%) (Figure 2). In patients undergoing endovascular coiling, those with poor functional outcomes tended to be older, have higher D-dimer levels, higher Hunt–Hess grades, GCS scores of 3–8, higher mFS grades, larger aneurysm volumes, were more likely to be located in the anterior cerebral artery and posterior circulation. Among patients undergoing surgical clipping, those with poor functional outcomes tended to be older, have higher Hunt–Hess grades, GCS scores of 3–8, higher mFS grades, higher D-dimer levels, and lower platelet counts (Tables 1, 2).

**Table 1:** Baseline characteristics of patients undergoing endovascular coiling.

Variables	Total (*n* = 638)	Good functional outcome (*n* = 542)	Poor functional outcome (*n* = 96)	*p*-value
Age, years	60.0 (52.0–68.0)	60.0 (51.8–67.0)	64.0 (53.0–72.0)	0.004
Gender				0.703
Female	413 (64.7%)	353 (65.1%)	60 (62.5%)	
Male	225 (35.3%)	189 (34.9%)	36 (37.5%)	
Hunt–Hess grade				<0.001
0–3	566 (88.7%)	514 (94.8%)	52 (54.2%)	
4–5	72 (11.3%)	28 (5.2%)	44 (45.8%)	
Hypertension				0.809
No	263 (41.2%)	225 (41.5%)	38 (39.6%)	
Yes	375 (58.8%)	317 (58.5%)	58 (60.4%)	
Diabetes mellitus				0.293
No	597 (93.6%)	510 (94.1%)	87 (90.6%)	
Yes	41 (6.4%)	32 (5.9%)	9 (9.4%)	
Body temperature, °C	36.8 (36.5–37.0)	36.8 (36.5–37.0)	36.8 (36.3–37.0)	0.068
MAP, mmHg	106.67 (96.67–117.33%)	106.67 (96.67–116.67%)	108.67 (98.67%–124.00%)	0.105
GCS score				<0.001
3–8	61 (9.6%)	19 (3.5%)	42 (43.8%)	
9–12	50 (7.8%)	35 (6.5%)	15 (15.6%)	
12–15	527 (82.6%)	488 (90.0%)	39 (40.6%)	
mFS				<0.001
0–2	245 (38.4%)	234 (43.2%)	11 (11.5%)	
3–4	393 (61.6%)	308 (56.8%)	85 (88.5%)	
Length, mm	4.0 (3.00–6.00)	4.0 (3.00–6.00)	4.58 (3.00–7.00)	0.063
Width, mm	4.0 (3.00–5.21)	4.0 (3.00–5.00)	4.5 (3.00–6.00)	0.191
Height, mm	4.0 (3.00–5.00)	3.88 (2.80–5.00)	4.00 (3.00–6.00)	0.038
Volume, mm^3^	33.51 (14.22–66.76)	31.42 (14.14–65.45)	41.89 (18.43–89.54)	0.027
Aneurysm location				0.018
Anterior cerebral artery	190 (29.8%)	154 (28.4%)	36 (37.5%)	
Middle cerebral artery	53 (8.3%)	49 (9.0%)	4 (4.2%)	
Internal carotid artery	135 (21.2%)	121 (22.3%)	14 (14.6%)	
Posterior communicating artery	200 (31.3%)	173 (31.9%)	27 (28.1%)	
Posterior circulation	60 (9.4%)	45 (8.3%)	15 (15.6%)	
Presence of hematoma				0.375
No	628 (98.4%)	535 (98.7%)	93 (96.9%)	
Yes	10 (1.6%)	7 (1.3%)	3 (3.1%)	
Use of stents				0.724
No	325 (50.9%)	274 (50.6%)	51 (53.1%)	
Yes	313 (49.1%)	268 (49.4%)	45 (46.9%)	
D-dimer, mg/L	0.81 (0.40–1.85)	0.72 (0.33–1.54)	1.86 (0.94–4.00)	<0.001
PLT, ×10^9^/L	208.0 (169.25–261.00)	207.5 (170.25–260.3)	211.50 (158.25–267.00)	0.858

MAP, mean arterial pressure; GCS, Glasgow Coma Scale; PLT, platelet.

**Table 2:** Baseline characteristics of patients undergoing neurosurgical clipping.

Variables	Total (*n* = 324)	Good functional outcome (*n* = 218)	Poor functional outcome (*n* = 106)	*p*-value
Age, years	60.0 (51.75–68.00)	58.5 (51.00–67.00)	63.0 (53.25–69.00)	0.020
Gender				0.720
Female	208 (64.2%)	138 (63.3%)	70 (66.0%)	
Male	116 (35.8%)	80 (36.7%)	36 (34.0%)	
Hunt–Hess grade				<0.001
0–3	200 (61.7%)	167 (76.6%)	33 (31.1.2%)	
4–5	124 (38.3%)	51 (23.4%)	73 (68.9%)	
Hypertension				1.000
No	91 (28.1%)	61 (28.0%)	30 (28.3%)	
Yes	233 (71.9%)	157 (72.0%)	76 (71.7%)	
Diabetes mellitus				1.000
No	308 (95.1%)	207 (95.0%)	101 (95.3%)	
Yes	16 (4.9%)	11 (5.0%)	5 (4.7%)	
Body temperature, °C	36.80 (36.5–37.0)	36.80 (36.5–37.0)	36.80 (36.5–37.1)	0.975
MAP, mmHg	111.00 (98.67–122.00)	111.00 (98.75–123.00)	111.50 (97.75–120.67)	0.741
GCS score				<0.001
3–8	95 (29.3%)	34 (15.6%)	61 (57.5%)	
9–12	23 (7.1%)	17 (7.8%)	6 (5.7%)	
12–15	206 (63.6%)	167 (76.6%)	39 (36.8%)	
mFS				<0.001
0–2	136 (42.0%)	121 (55.5%)	15 (14.2%)	
3–4	188 (58.0%)	97 (44.5%)	91 (85.8%)	
Length, mm	4.00 (3.00–6.00)	4.00 (3.00–5.20)	4.00 (3.00–6.00)	0.651
Width, mm	4.00 (3.00–5.00)	4.00 (3.00–5.00)	4.00 (3.00–5.00)	0.088
Height, mm	4.00 (3.00–5.00)	4.00 (3.00–5.00)	4.00 (3.00–5.00)	0.093
Aneurysm volume, mm^3^	33.51 (14.14–65.45)	31.42 (14.14–62.83)	33.51 (14.14–89.54)	0.186
Aneurysm location				0.231
Anterior cerebral artery	106 (32.7%)	75 (34.4%)	31 (29.2%)	
Middle cerebral artery	126 (38.9%)	81 (37.2%)	45 (42.5%)	
Internal carotid artery	40 (12.3%)	26 (11.9%)	14 (13.2%)	
Posterior communicating artery	44 (13.6%)	33 (15.1%)	11 (10.4%)	
Posterior circulation	8 (2.5%)	3 (1.4%)	5 (4.7%)	
Presence of hematoma				0.791
No	276 (85.2%)	187 (85.8%)	89 (84.0%)	
Yes	48 (14.8%)	31 (14.2%)	17 (16.0%)	
D-dimer, mg/L	1.64 (0.73–2.93)	1.33 (0.66–2.48)	2.33 (1.34–3.99)	<0.001
PLT, ×10^9^/L	260.00 (160.75–349.25)	286.00 (192.75–362.50)	194.50 (119.25–291.75)	<0.001

MAP, mean arterial pressure; GCS, Glasgow Coma Scale; PLT, platelet.

### Univariate and multivariate logistic regression analysis

**Figure 3:**
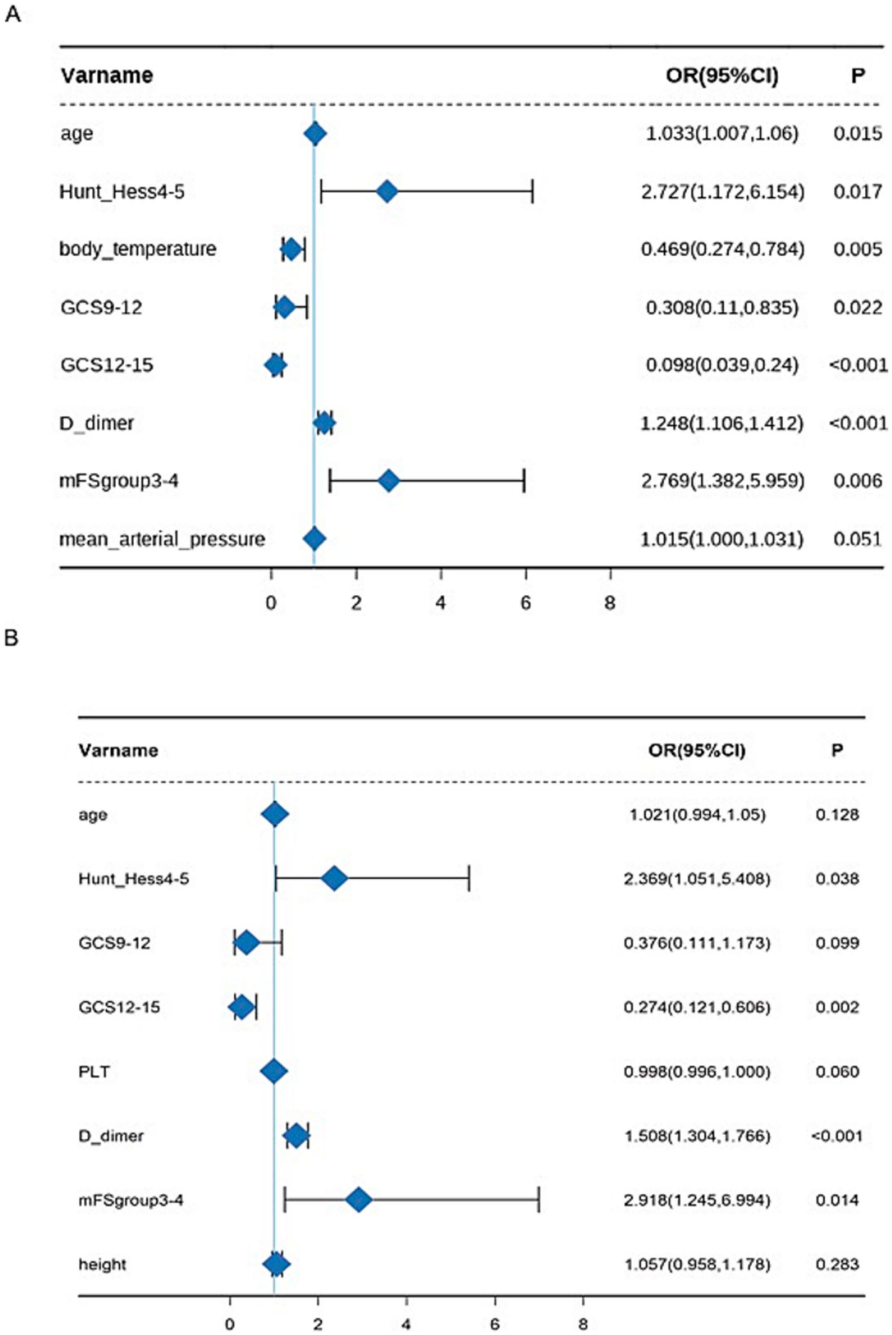
Forest plot based on multivariate analysis in the endovascular coiling **(A)** and surgical clipping groups **(B)**.

We utilized univariate and multivariate logistic regression models to assess the impact of clinical variables and scores on outcomes. The results revealed that, in the endovascular coiling group, age [adjusted odds ratio (OR): 1.033, 95% confidence interval (CI): 1.007–1.06, *p* = 0.015], Hunt–Hess grade (adjusted OR: 2.727, 95% CI: 1.172–6.154, *p* = 0.017), GCS score (9–12: adjusted OR: 0.308, 95% CI: 0.11–0.835, *p* = 0.022; 12–15: adjusted OR: 0.098, 95% CI: 0.039–0.24, *p* < 0.001), mFS (adjusted OR: 2.769, 95% CI: 1.382–5.959, *p* = 0.006), D-dimer (adjusted OR: 1.248, 95% CI: 1.106–1.412, *p* < 0.001), and body temperature (adjusted OR: 0.469, 95% CI: 0.274–0.784, *p* = 0.005) were significantly associated with early adverse functional outcomes (Table 3). Whereas, in the surgical clipping group, Hunt–Hess grade (adjusted OR: 2.369, 95% CI: 1.051–5.408, *p* = 0.038), GCS score (12–15: adjusted OR: 0.274, 95% CI: 0.121–0.606, *p* = 0.002), mFS (adjusted OR: 2.918, 95% CI:1.245–6.994, *p* = 0.014), and D-dimer (adjusted OR: 1.508, 95% CI: 1.304–1.766, *p* < 0.001) were independent predictors of early functional outcomes (Table 4). The forest plot displays the significant multivariable factors (Figures 3A,B).

**Table 3:** Logistic analysis of early functional outcomes after endovascular coiling.

Variables	Univariable^a^		Multivariable^b^	
	OR (95% CI)	*p-*value	OR (95% CI)	*p-*value
Sex				
Female				
Male	1.121 (0.71–1.748)	0.619		
Age	1.031 (1.01–1.053)	0.004	1.033 (1.007–1.06)	0.015
Hunt–Hess				
0–3				
4–5	15.533 (9.002–27.292)	<0.001	2.727 (1.172–6.154)	0.017
Body temperature	0.613 (0.379–0.979)	0.043	0.469 (0.274–0.784)	0.005
GCS				
3–8				
9–12	0.194 (0.084–0.429)	<0.001	0.308 (0.11–0.835)	0.022
12–15	0.036 (0.019–0.067)	<0.001	0.098 (0.039–0.24)	<0.001
PLT	0.999 (0.996–1.002)	0.574		
D-dimer	1.375 (1.248–1.522)	<0.001	1.248 (1.106–1.412)	<0.001
mFS				
0–2				
3–4	5.871 (3.193–11.875)	<0.001	2.769 (1.382–5.959)	0.006
Hypertension				
No				
Yes	1.083 (0.698–1.698)	0.723		
Diabetes				
No				
Yes	1.649 (0.719–3.44)	0.205		
Length	1.032 (0.976–1.086)	0.239		
Width	1.018 (0.952–1.08)	0.565		
Height	1.044 (0.966–1.12)	0.252		
Volume	1 (0.999–1)	0.985		
MAP	1.014 (1.001–1.027)	0.028	1.015 (1.000–1.031)	0.051
Stent				
No				
Yes	0.902 (0.583–1.393)	0.642		
Aneurysm location				
Anterior cerebral artery				
Middle cerebral artery	0.349 (0.101–0.928)	0.057		
Internal carotid artery	0.495 (0.248–0.94)	0.037		
Posterior communicating artery	0.668 (0.385–1.147)	0.146		
Posterior circulation	1.426 (0.702–2.8)	0.312		

a Univariable logistic regression models.

b Multivariable logistic regression model adjusting for age at diagnosis Hunt–Hess grade, body temperature, MAP, GCS score, and D-dimer.

OR, odd ratio; CI, confidence interval; MAP, mean arterial pressure; GCS, Glasgow Coma Scale.

**Table 4:** Logistic analysis of early functional outcomes after surgical clipping.

Variables	Univariable^a^		Multivariable^b^	
	OR (95% CI)	*p-*value	OR (95% CI)	*p-*value
Sex				
Female				
Male	0.887 (0.542–1.439)	0.630		
Age	1.026 (1.003–1.049)	0.027	1.021 (0.994–1.05)	0.128
Hunt–Hess				
0–3				
4–5	7.244 (4.358–12.281)	<0.001	2.369 (1.051–5.408)	0.038
Body temperature	1.025 (0.644–1.613)	0.916		
GCS				
3–8				
9–12	0.197 (0.066–0.522)	0.002	0.376 (0.111–1.173)	0.099
12–15	0.13 (0.075–0.223)	<0.001	0.274 (0.121–0.606)	0.002
PLT	0.997 (0.995–0.999)	0.001	0.998 (0.996–1.000)	0.060
D-dimer	1.266 (1.132–1.423)	<0.001	1.508 (1.304–1.766)	<0.001
mFS				
0–2				
3–4	7.568 (4.228–14.364)	<0.001	2.918 (1.245–6.994)	0.014
Hypertension				
No				
Yes	0.984 (0.591–1.662)	0.952		
Diabetes				
No				
Yes	0.932 (0.287–2.635)	0.898		
Length	1.058 (0.994–1.128)	0.076		
Width	1.064 (0.991–1.151)	0.095		
Height	1.09 (1.004–1.197)	0.049	1.057 (0.958–1.178)	0.283
Volume	1 (1.000–1.001)	0.284		
MAP	0.994 (0.981–1.007)	0.360		
Aneurysm volume	1 (1–1.001)	0.284		
Anterior cerebral artery				
Middle cerebral artery	1.344 (0.774–2.354)	0.296		
Internal carotid artery	1.303 (0.592–2.801)	0.502		
Posterior communicating artery	0.806 (0.351–1.761)	0.598		
Posterior circulation	4.032 (0.933–20.641)	0.067		

a Univariable logistic regression models.

b Multivariable logistic regression model adjusting for age at diagnosis, blood group, GCS score, aneurysm width, aneurysm height, D-dimer, and PLT.

OR, odd ratio; CI, confidence interval; MAP, mean arterial pressure; GCS, Glasgow Coma Scale.

### The relative weights of impact on the early functional outcome

**Figure 4:**
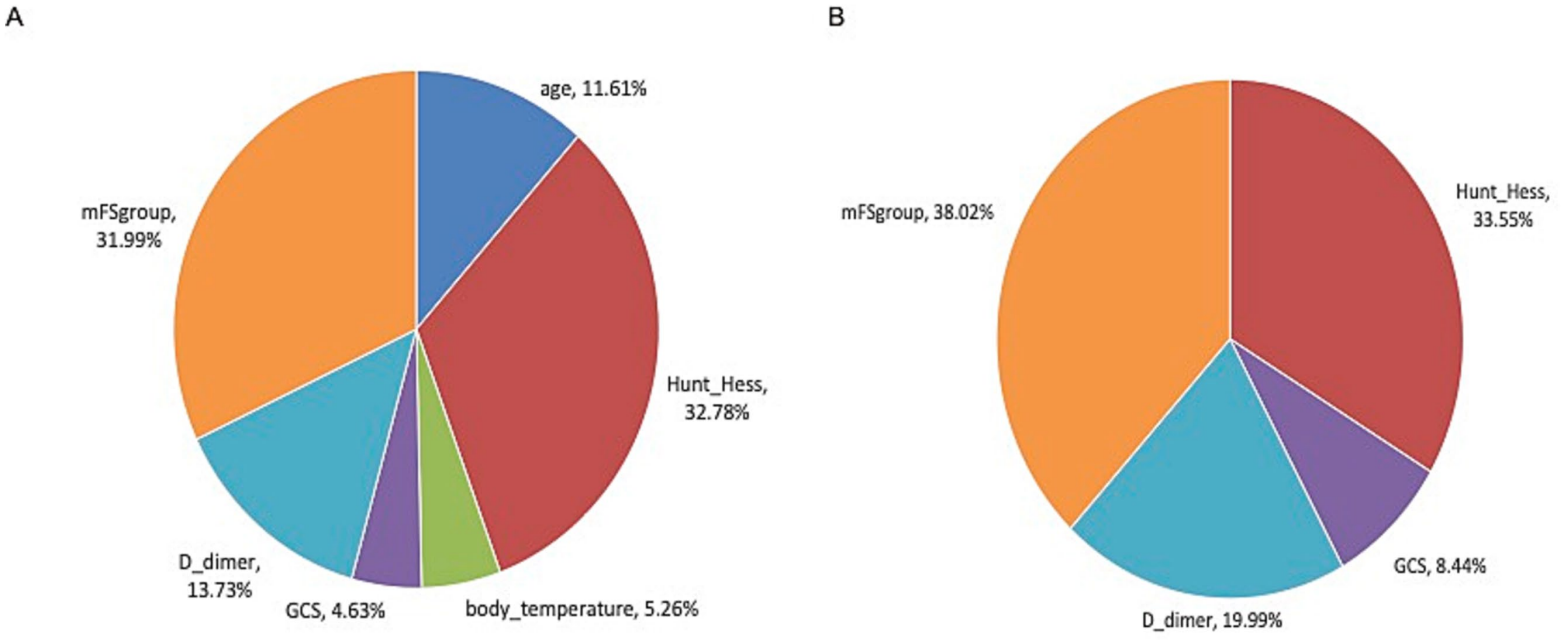
The relative weights of variables influencing early functional outcomes in the endovascular coiling **(A)** and surgical clipping groups **(B)**.

We further assessed the relative impact of significant variables on early functional outcomes using multivariate logistic regression analysis. In the endovascular coiling group, the proportions of significant variables were as follows: Hunt–Hess grade contributed 32.78%, mFS contributed 31.99%, D-dimer contributed 13.73%, age contributed 11.61%, body temperature contributed 5.26%, and GCS contributed 4.63%. In the surgical clipping group, mFS contributed 38.02%, Hunt–Hess grade contributed 33.55%, D-dimer contributed 19.99%, and GCS contributed 8.44% (Figures 4A,B).

### Nomogram for early functional outcome and validation

**Figure 5:**
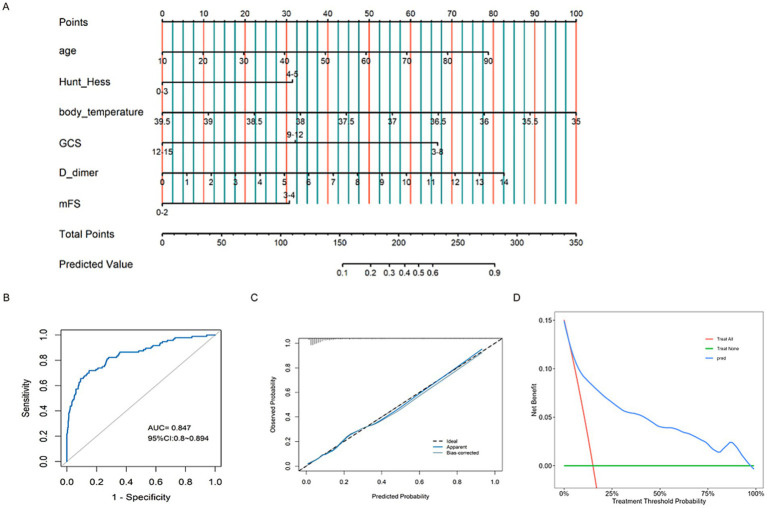
Constructing a nomogram **(A)** to forecast functional outcomes in patients undergoing endovascular coiling, and validating it through ROC curves **(B)**, calibration plots **(C)**, and DCA curves **(D)**.

**Figure 6:**
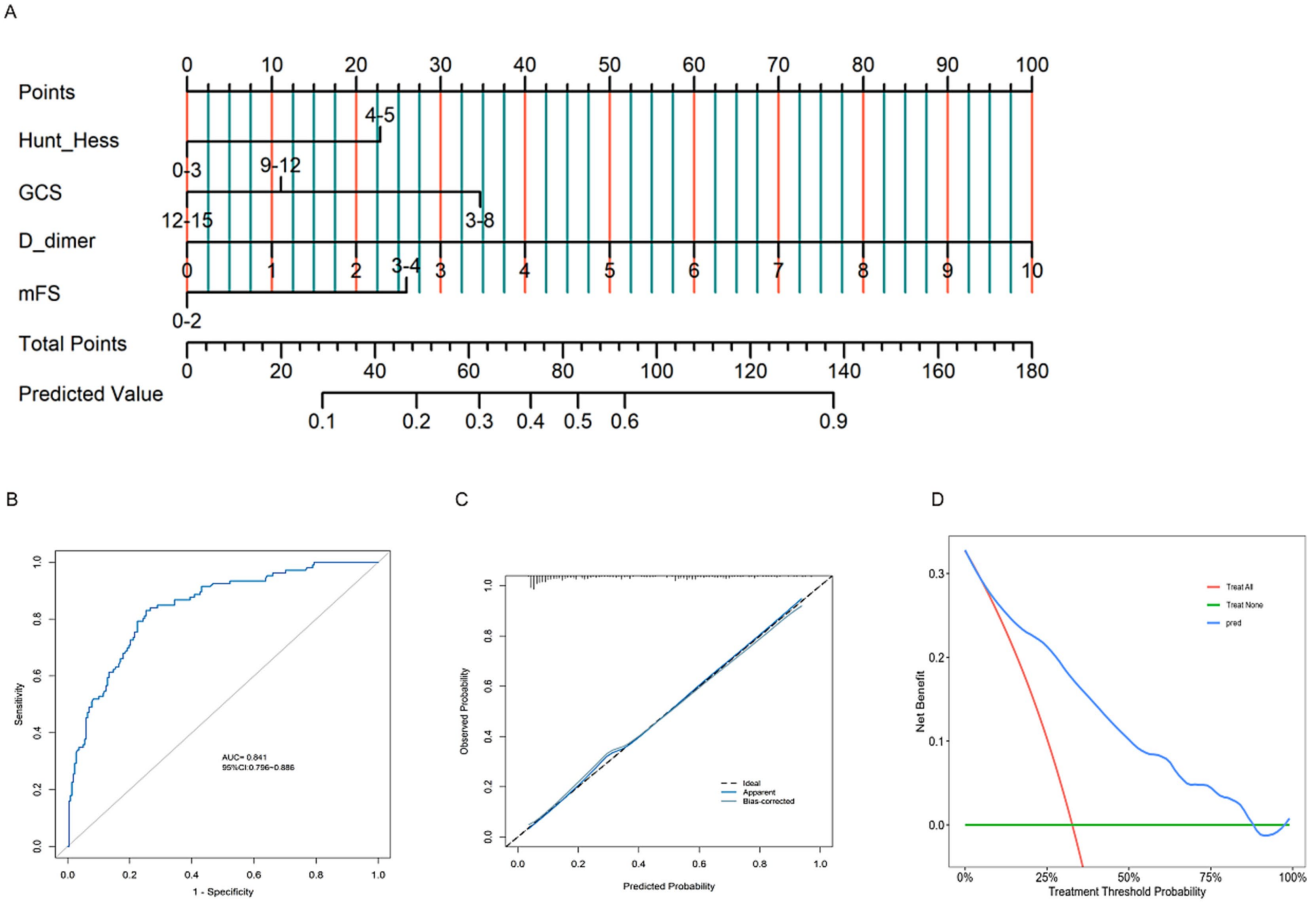
Constructing a nomogram **(A)** to forecast functional outcomes in patients undergoing surgical clipping, and validating it through ROC curves **(B)**, calibration plots **(C)**, and DCA curves **(D)**.

**Figure 7:**
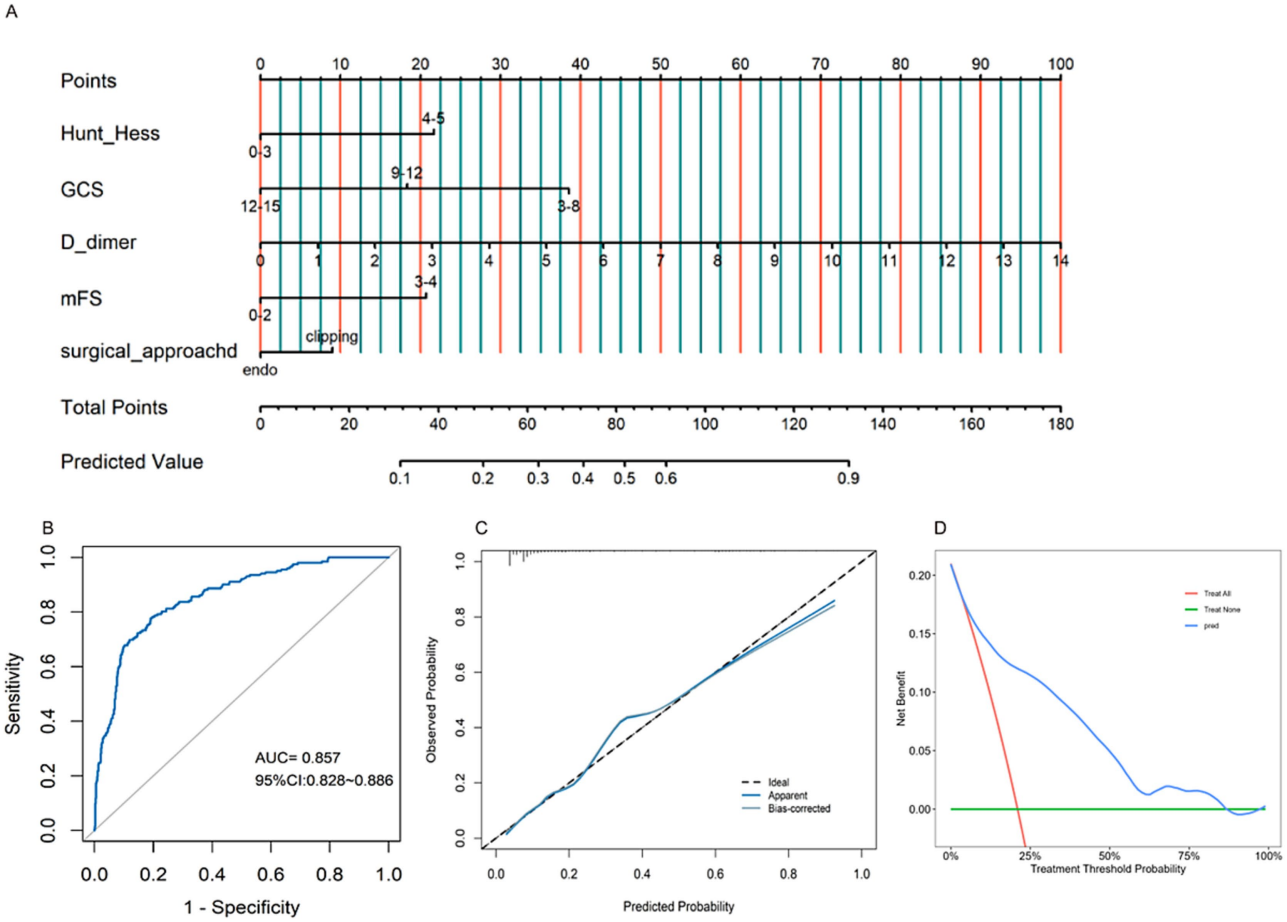
Constructing an integrated nomogram **(A)** to forecast functional outcomes of aSAH patients after surgery, and validating it through ROC curves **(B)**, calibration plots **(C)**, and DCA curves **(D)**.

Nomogram models were developed for different groups based on early functional outcomes (Figures 5A, 6A). In the endovascular coiling group, the ROC curve indicated good predictive accuracy, with an AUC value of 0.847 (95% CI: 0.800–0.894) (Figure 5B), and the calibration curve demonstrated close alignment between the model and predictions (Figure 5C). The decision curve analysis (DCA) curve showed good clinical predictive value across different threshold probabilities (Figure 5D). Similarly, in the surgical clipping group, the ROC curve showed good predictive accuracy, with an AUC value of 0.841 (95% CI: 0.796–0.886) (Figure 6B), and the calibration curve exhibited basic alignment with predictions (Figure 6C). The DCA curve also demonstrated good clinical predictive value across different threshold probabilities (Figure 6D). In addition, by incorporating the surgical approach as an additional variable into the shared predictive factors, we developed a new, integrated nomogram model (Figure 7A). This model achieved an AUC of 0.857 for predicting poor outcomes within 1 month (Figure 7B). The calibration curve demonstrated good agreement between predicted and observed outcomes, and the DCA showed favorable clinical utility across a range of threshold probabilities (Figures 7C,D).

### Nomogram-based risk stratification predicts early functional outcome

Based on the risk scores predicted by the nomogram, patients were categorized into three groups: low risk, medium risk, and high risk. In the endovascular coiling group, individuals in the medium-risk group faced a 2.791-fold higher risk compared to those in the low-risk category, while those in the high-risk group experienced a significantly elevated risk of 17.506 times compared to the low-risk group (Table 5). Similarly, within the surgical clipping group, participants classified in the medium-risk tier were at a 5.291-fold increased risk relative to the low-risk counterparts, with those in the high-risk category encountering an even greater risk, elevated by a substantial 26.641 times compared to the low-risk group (Table 5). These differences were statistically significant, highlighting the clinical significance of our nomogram model in integrating clinical scores and parameters.

**Table 5:** Nomogram risk stratification.

Variables	Endovascular coiling		Surgical clipping	
	OR (95% CI)	*p-*value	OR (95% CI)	*p-*value
Low-risk group				
Medium-risk group	2.791 (1.134, 7.868)	0.034	5.291 (2.326, 13.671)	<0.001
High-risk group	17.506 (8.014, 46.109)	<0.001	26.641 (11.913, 68.537)	<0.001

## Discussion

Despite significant advancements in managing patients with subarachnoid hemorrhage, the mortality rate remains high. The case-fatality rate for aSAH ranges from 32 to 67%, with approximately one-third of survivors experiencing long-term disability or cognitive impairment (11). Numerous studies have identified risk factors that influence the onset and progression of aSAH, including sex, hypertension, alcohol consumption, and smoking (12–14), as well as aneurysm-related factors such as size and location (15–17), influence the occurrence and development of aSAH. In addition, proteomic analyses have revealed that proteins involved in the focal adhesion and extracellular matrix–receptor interaction pathways may play a key role in the pathogenesis of intracranial aneurysms (18). Currently, endovascular coiling and neurosurgical clipping have become the main treatment methods for aSAH.

In our study, the incidence of early adverse functional outcomes was significantly lower in patients undergoing endovascular coiling compared to those treated with neurosurgical clipping, which is consistent with current clinical guidelines (19, 20). However, endovascular coiling is associated with increased hospitalization costs (21), and carries certain risks, including a lower rate of complete aneurysm occlusion and a higher risk of delayed rebleeding (22–24). Based on this, we have established two new nomogram models, which were based on commonly used clinical scoring systems (such as the Hunt–Hess grading scale and the GCS) and some clinical parameters, to evaluate the early functional outcomes of these two different surgical methods. Each variable corresponds to a score on the top line of the nomogram. The total score, calculated by summing the scores of all variables, is projected onto the bottom scale to estimate the probability of early postoperative poor outcomes in patients with aSAH (25). Moreover, our results indicate that the prognostic models for both surgical approaches demonstrate good discriminatory power and calibration. Therefore, this tool has the potential to assist neurosurgeons in clinical decision-making and enhance the effective communication of prognostic information to patients’ families. In addition, based on the shared independent predictors identified from the two models, we further developed an integrated nomogram incorporating the surgical approach as a covariate. This comprehensive model also demonstrated favorable predictive performance, supporting its potential utility in clinical practice for holistic risk stratification and management planning.

This study found that among patients treated with endovascular coiling, those who exhibited poorer early functional outcomes typically had older age, higher mFS scores, higher Hunt–Hess grades, and lower GCS scores, consistent with prior research (26, 27). Additionally, we observed that these patients often presented with elevated D-dimer levels, larger aneurysm sizes, and anterior cerebral artery location of the aneurysm. In patients undergoing surgical clipping, factors influencing early functional outcomes typically included Hunt–Hess and mFS scores, which align with findings from Li’s et al. (21) study. Furthermore, our study indicated that lower GCS scores, elevated D-dimer levels, and decreased platelet counts were predictive of poorer early functional outcomes. Our results highlight the different perioperative priorities associated with each surgical modality. For high-risk patients undergoing endovascular treatment, enhanced monitoring for thromboembolic events and proactive temperature management may be warranted. Surgical candidates with elevated D-dimer levels may benefit from preoperative optimization of hemostasis and postoperative antifibrinolytic therapy.

Through multifactorial logistic analysis, age, body temperature, D-dimer, mFS score, Hunt–Hess grade, and GCS score were identified as independent risk factors for early functional outcomes in the endovascular coiling group. For surgical clipping treatment, Hunt–Hess grade, GCS score, mFS, and D-dimer level were determined as independent risk factors influencing early functional outcomes. Of note, the relative contribution analysis revealed that Hunt–Hess grade and mFS were the dominant predictors in both models, while D-dimer ranked among the top three contributors in each group, further supporting its clinical relevance. Interestingly, D-dimer levels can predict outcomes in both surgical methods, possibly because: (1) in neurosurgical procedures, stress may affect patients’ hemostatic and fibrinolytic functions; (2) changes in D-dimer levels can reflect alterations in coagulation status, and abnormal D-dimer levels may indicate postoperative bleeding and thrombotic complications (28–30). Additionally, hypothermia may lead to poorer outcomes in patients undergoing endovascular coiling treatment, possibly due to its adverse effects on resuscitation and potential myocardial damage (31). For the first time, we have identified that D-dimer levels significantly influence early functional outcomes in patients treated with either endovascular coiling or surgical clipping. This finding place D-dimer levels second only to Hunt–Hess grading and the mFS scoring system, surpassing GCS scores in importance. Moreover, the inclusion of D-dimer challenges traditional reliance on clinical grading scales alone. Implementing these models could improve resource allocation by identifying patients who may derive greater benefit from intensive care or targeted interventions.

The study has some limitations. Firstly, being a retrospective study, there may be confounding factors affecting the stability of the results. Secondly, the assessment of early functional outcomes was conducted only one-month post-surgery, requiring further long-term follow-up to evaluate more comprehensive outcomes. Thirdly, we did not include emerging biomarkers or advanced imaging features, which could potentially enhance predictive accuracy. Therefore, prospective studies with longer follow-up periods are needed to validate these findings and explore intervention strategies based on these risk profiles.

## Data Availability

The raw data supporting the conclusions of this article will be made available by the authors, without undue reservation.
